# Contribution of Trunk Rotation and Abdominal Muscles to Sprint Kayak Performance

**DOI:** 10.5114/jhk/169939

**Published:** 2023-11-28

**Authors:** Mathew B. Brown, Russell Peters, . Lauder Mike A

**Affiliations:** 1 School of Psychology and Life Sciences, Canterbury Christ Church University, Canterbury, UK.; 2 Department of Sport and Health, Newman University, Birmingham, UK.; 3 Chichester Institute of Sport, Nursing and Allied Health, University of Chichester, Chichester, UK.

**Keywords:** canoe, electromyography, elite, kinetics

## Abstract

Over the past two decades the importance of trunk contribution to sporting performance has been highlighted through the expanse of literature concerning core stability and strength. However, the role of trunk motion and the abdominal muscles are yet to be established during sprint kayak performance. The purpose of this study was to determine the associations among trunk rotation, kayak velocity, and abdominal muscle activity during on-water sprint kayaking. Eight international paddlers completed five 150 m sprint trials. During each trial peak muscle activation (peak root-mean-squared electromyogram) of the latissimus dorsi, rectus abdominus, external obliques and rectus femoris for ipsilateral (stroke side) and contralateral (opposite side) were recorded as the paddler passed through a 5-m calibrated volume, in conjunction with upper and lower trunk rotation and kayak velocity. Results indicated a significant strong negative relationship between lower trunk rotation and peak velocity (r = −0.684, p < 0.05). Furthermore, a significant strong positive relationship (p < 0.05) with mean velocity was identified for the contralateral rectus abdominus and multiple significant associations between the rectus femoris, rectus abdominus and external obliques during the paddle stroke. Findings indicate that limiting the rotation of the lower trunk will increase both the peak and the mean velocity, with the rectus abdominus, external oblique and rectus femoris combining to assist in this process. Training should therefore focus on developing the strength of these muscle groups to enhance performance.

## Introduction

Sprint kayaking success is determined by average velocity over a given distance and has been investigated from a variety of approaches (Li, 2017). Predominantly, biomechanical assessment has been conducted using kinematics (Baker et al., 1999; Kendal and Sanders, 1992; Lopez Lopez and Ribas Serna, 2011; Sanders and Kendal, 1992) and kinetics (Aitken and Neal, 1992; Gomes et al., 2015; Mononen et al., 1995; Mononen and Viitasalo, 1995; Petrovic et al., 2021; OnariciGüngöret al., 2023), with attention of the research centring on the upper limbs and the paddle. Focus upon force production and the motion of the paddle, resulting from occlusion of the lower limbs by the kayak shell and the significant association between the magnitude of paddle force and average velocity (Mononen et al., 1995; Mononen and Viitasalo, 1995),means the contribution of the legs and the trunk is less well understood.

A former Olympic medallist and international coach, ImreKemecsey, has indicated that the technique required to succeed at the highest level requires contribution from the trunk and legs in addition to the motions of the paddle and arms (Kemecsey, 1986). This proposition has found empirical support in the peer reviewed research, as Petrone et al. (2006) identified that increases in trunk rotation, during ergometer paddling, through the use of a rotating seat, increased paddle force production (fixed seat: 320 N, rotating seat 465 N). Moreover, Bjerkefors et al. (2018) identified significant increases in peak trunk rotation and trunk range of motion (RoM) during higher intensity paddling. Furthermore, Brown et al. (2010, 2011) supported the importance of the trunk, through both notational analysis and electromyographic investigation. Notational analysis highlighted that significantly greater trunk rotation and leg motion was incorporated within the techniques employed by international level paddlers, in comparison to national and club level paddlers (Brown et al., 2011). Moreover, Brown et al. (2010) identified moderate to strong (*r*> 0.65) significant associations between peak trunk muscle activation and peak and mean paddle force during on water paddling; supported by sporadic significant findings between paddle force and leg musculature.

Departing from the proposed (Kemecsey, 1986), and somewhat supported (Bjerkefors et al., 2018; Brown et al., 2010, 2011; Petrone et al., 2006) role of the trunk muscles in force production, a secondary role in paddling performance can be suggested. Previous studies have identified that a stable trunk during a seated position has a significant impact on force production in both the lower and the upper limbs (Hart et al., 1984; Kebaetse et al., 1999). Similarly, the seated position adopted during paddling results in the trunk forming the support structure around which the arms/paddle motion is produced (Li, 2017). This role could be of greater importance when the natural instability of the kayak is considered, as the design of the sprint kayak has evolved to maximise velocity by reducing the form drag (Robinson et al., 2002), directly affecting the lateral stability of the kayak. Consequently, the paddler is required to simultaneously balance the kayak, while maintaining the highest average kayak velocity, indicating that stability in the trunk is important. Furthermore, research in other dynamic sports has identified the natural stabilising role of core muscles of the trunk (Kibler et al., 2006) during various sporting motions (running, throwing and kicking). While this has not yet been considered directly during sprint kayaking, a sport in which the competitive environment is highly unstable, work undertaken by Davidek et al. (2018) indicated that a 6-week period of dynamic neuromuscular stabilization, focusing on core stability, significantly increased paddling force production. This highlights limitations in the assertions promoted by Petrone et al.’s (2006) research which indicated that greater trunk rotation resulted in greater force and consequently, velocity (Mononen et al., 1995; Mononen and Viitasalo, 1995). However, increased rotation has not been demonstrated to directly affect boat velocity, as findings were attained from on ergometer testing. Furthermore, Petrone et al. (2006) gave no consideration of the effects of the extra rotation on the wetted surface area and the resulting drag experienced during paddling; the most restrictive factor to velocity in water-based activities (Pendergast et al., 2003). Therefore, although previous researchers have attempted to determine the importance of the trunk motion and its muscular activity in paddling performance, the question of their contribution to kayak velocity is still unanswered (Brown et al., 2010; Pertrone et al., 2006).

Previous literature (Bjerkefors et al., 2018; Brown et al., 2010, 2011; Kemecsey, 1986; Li, 2017; Petrone et al., 2006)has indicated that the trunk is important in kayaking performance, however, the exact nature of the contribution to boat velocity has not been established, as many researchers used secondary measures of performance (force production). Derived from previous work, it was hypothesised that increases in the rectus abdominus, external obliqus, and latissimus dorsi activation would exhibit significant associations with increases in kayak velocity. Furthermore, it was hypothesised that increases in trunk rotations would demonstrate significant positive associations with kayak velocity.

## Methods

### Participants

Eight elite level flat water sprint kayakers, male (n = 6) and female (n = 2), aged 24.6 ± 4.3 years old, all competing at the international level, volunteered to complete the testing protocol. Prior to inclusion all participants had the experimental procedures outlined and completed medical screening questionnaires as well as informed consent. All testing protocols were approved by the local institutional review board (University College Chichester, protocol code 2223_39) prior to the commencement of testing and all testing was carried out in accordance with the Declaration of Helsinki.

### Design and Procedures

Each participant was prepared following SENIAM guidelines (SENIAM n.d.) with passive surface electrodes (Ambu® blue sensor T) spaced at 0.05 m over the belly of the left and right external oblique, rectus abdominus, rectus femoris, and latissimus dorsi. The external oblique sensors were positioned above the anterior superior iliac spine, halfway between the anterior superior iliac spine and the 12^th^ rib; orientated in the direction of the muscle fibres. Sensors recording the activation in the rectus abdominus were positioned in a vertical orientation, 0.02 m lateral to the umbilicus. Sensors for the rectus femoris were positioned 50% distally on the line from the anterior spina iliac superior to the superior aspect of the patella. The sensors on the latissimus dorsi were orientated at a slight oblique angle, two thirds of the way from the spine to the lateral edge of the body, 0.04 m below the inferior tip of the scapula. All signals were amplified using MIE Medical Research Ltd 4K preamplifiers (3–250 Hz), while miniature MIE data loggers sampling at 500Hz were utilised for data storage.

Participants were fitted with two single-axis waterproof torsiometers (Biometrics Ltd. Model number Q150/W) positioned on the posterior of the trunk over the spineous processes to measure relative axial rotation in the upper and lower trunk from a neutral seated position. The lower trunk torsiometer was positioned over vertebrae L5 to T10/11 and the upper trunk torsiometer was positioned over T9 to T1/C7 (dependent on the length of the individual participant spinal column). Torsiometers were attached with double sided tape at each end point and reinforced with medical tape. Alternative methods of attachment similar to Burnett et al. (2008) were investigated, however, due to the positioning of the upper trunk torsiometer, this approach was not functional as the motion of the scapulae and increased musculature in the highly developed trapezius and latissimus dorsi of the testing population, would have exaggerated motion of the attachment points reducing the accuracy of the rotational measures. The same attachment method was used for both torsiometers to maintain consistency. The accuracy of the torsiometers employed has been identified, in relation to radiographic measurements, as 2.3º ± 2.2º (Bible et al., 2010; Boocock et al., 1994). Data were sampled at 500 Hz and stored using miniature MIE data loggers. Following preparation, each participant completed a series of maximal voluntary contractions (MVCs) against manual resistance. Each maximal voluntary contraction (MVC) was conducted for three seconds and repeated five times, from which peak activation over a 0.5-s window was determined to be maximal activation. The MVC procedures for each muscle were as follows:

*Rectus abdominus*:participants stood upright with the posterior abdominal surface pressed against a wall. Participants were instructed to produce a forward crunch motion against which two researchers applied resistance at the shoulder to ensure no motion would take place.

*External oblique*: participants stood upright with the posterior abdominal surface pressed against a wall. Participants were instructed to produce a diagonal crunch motion against which two researchers applied resistance at the shoulders to ensure no motion would take place. This was repeated for each side.

*Rectus Femoris*: participants were seated with their back firmly pressed into the back of a seat with the hip and knee positioned at 90°. Participants were instructed to attempt to extend the knee, without rotating the thigh, against manual resistance applied upon the leg above the ankle. This was repeated for each side.

*Latissimus Dorsi*: participants stood upright, abducted both shoulders by 90° and flexed the elbows by 90°. Researchers positioned their shoulders under the elbows of participants and clasped their hand across the top of the shoulder to resist any upward motion. Participants were instructed to mimic a latissimus dorsi pull down motion against the resistance provided by researchers.

Paddle stroke kinetic variables were measured using strain gauges (Sperlich and Sperlich, Germany) mounted on the paddle shaft midway between the grip position and the connection between the paddle shaft and the blade. The strain gauges were orientated perpendicular to the blade surface, mounted out of the phase from one another by 60–85°, dependent on the feathering angle of the blades on the participant’s paddle. Calibration was completed before and after data collection, ensuring no significant alterations occurred during paddling, using a 196.2 N weight.

Once prepared, participants were encouraged to adopt their natural paddling position using their own kayak and paddle. Participants then completed a standard on-water warm up consisting of 1000-m sub-maximal paddling, which also allowed for the paddler to habituate to the electrodes, torsiometers and monitoring equipment carried on the kayak. Following the warm up, paddlers completed five trials, each consisting of 150 m maximal sprints through a calibrated volume (5 x 2.5 x 1.8 m) 100 m into the trial. As the participant passed through the calibrated volume, two HSC-200PM cameras (Peak Performance Technologies Ltd.) captured the video footage at 200 Hz, using two SVHS video recorders (Panasonic, AG MD830), allowing calculation of mean velocity and peak intra stroke kayak velocity. The whole trial was captured using a digital video camera (Sony, DCR-PC53E) at 60 Hz to assist in post testing data synchronisation.

### Statistical Analysis

Data synchronisation was conducted in two phases; firstly, an electrical impulse which caused a saturation spike in the data traces for the electromyography (EMG) and torsiometer data was used before the commencement of the trials, following completion of the warm up. Participants were instructed to sit in a neutral position for 5 s with the paddle resting across the cockpit and their hands in their usual grip; this allowed for establishing a zero point for each of the torsiometers. This position is characterised with the participant seated in their kayak, trunk held in the upright position (akin to the paddling position), legs flexed at the hip, knees slightly bent, and feet resting on the footplate. The exact angular position was not assessable and may have varied moderately based on anthropometrics of the participant and the kayak set up. All spinal rotations are reported as relative rotations towards the stroke side (ipsilateral shoulder moving posteriorly) during the pull phase with reference to the neutral position. The second phase of synchronisation, between the kinematic and datalogger systems was completed using the digital footage of the full trial. Identification of the specific stroke coinciding with the calibrated volume, to allow the determination of associated muscle activations and trunk rotations from the data logger, was accomplished by stroke counting from the start of the trial.

Following data synchronisation, a five second section was extracted, coinciding with the participant moving through the calibrated volume, reconstruction accuracy of which was 0.4 ± 0.01%. Video footage was reconstructed using Peak Motus 32 (Vicon Motion Systems) analysis software and smoothed using a 4^th^ order Butterworth filter with a 6 Hz cut off, allowing calculation of mean kayak velocity and peak intra stroke kayak velocity during the pull phase for the left and the right stroke from a 6^th^ order polynomial of the x component of the centroid of the cranium. EMG traces were downloaded to MyoDat software (v.6, MIE Medical Research Ltd., UK) and conditioned using a root mean squared linear envelope (50 ms window) and normalised to MVC. Peak activation (PA) and mean activation (MA), normalised root-mean-squared EMG’s (%MVC), for each stroke were extracted for all muscles during the pull phase of the left and right strokes. Torsiometer data were normalised to a baseline value extracted from the central 3 s recorded while the participant was seated in the neutral position prior to testing, allowing extraction of peak trunk rotations and the range of trunk rotation throughout the stroke. Data collected from muscles on both left and right sides were classified as either ipsilateral (the same side as the active stroke) or contralateral (the opposite side to the active stroke) muscle groups for the purpose of analysis.

Stroke efficiency was calculated by dividing mean stroke force by peak intra stroke force (Gomes et al., 2015) and the rate of force development was estimated by dividing peak force by absolute time to peak force. Following tests for normality (Shapiro-Wilk), Pearson’s and Spearman’s correlation coefficients were calculated with significance set at the standard alpha level (0.05), when appropriate, for the relationships between peak RMS EMG, trunk motion and velocity variables. All statistical analyses were conducted using IBM SPSS (version 24, IBM). Correlation strength was characterised as 0.0 to ± 0.299 none/very weak, ± 0.300 to ±0.499 weak, ± 0.500 to ± 0.699 moderate and ± 0.700 to ± 1.0 strong.

## Results

Mean kayak velocity produced by the group was recorded at 4.78 ± 0.43 m·s^−1^, which was comparable to the average velocity (4.79 ± 0.07 m·s^−1^) of finalists in the men’s K1 1000 m world cup events through 2017–18. Furthermore, peak intra stroke velocities (left 5.25 ± 0.17 m·s^−1^; right 5.23 ± 0.19 m·s^−1^) were within ranges previously identified within the literature (Baker et al., 1999; Hay and Kaya, 1998; Kendal and Sanders, 1992) (Table 1).

**Table 1:** Descriptive statistics for kinetic, velocity and trunk rotation measures.

	Mean	SD	SE	n	Confidence Interval	
					Lower Bnd.	Upper Bnd.
Impulse (Ns)	92.95	9.55	2.73	16	87.86	98.04
Peak Velocity (m·s^−1^)	5.24	0.49	0.13	16	4.98	5.50
Mean Velocity (m·s^−1^)	4.78	0.43	0.11	16	4.54	5.01
Stroke Efficiency	0.63	0.04	0.01	16	0.61	0.65
Rate of Force Production (N·s^−1^)	1971.3	359.1	89.8	16	1795.3	2147.3
Upper Trunk Rotation to the Stroke Side (^o^)	11.72	5.13	1.36	16	8.99	14.45
Lower Trunk rotation to the Stroke Side (^o^)	12.07	6.22	1.66	14	8.48	15.66

The rate of force development demonstrated moderate positive significant association with both mean and peak kayak velocity (*r* = 0.645, *p*< 0.05 and *r* = 0.656, *p*< 0.05, respectively; Table 2). Trunk rotation in the upper trunk was characterised by a mean maximal ipsilateral rotation of 11.7 ± 5.1° and maximal lower trunk rotation was 12.1 ± 6.2°. Lower trunk rotation to the stroke side displayed moderate significant negative correlations with both mean (*r* = −0.567, *p*< 0.05) and peak (*r* = −0.684, *p*< 0.01) velocity.

**Table 2:** Correlations between velocity and trunk rotations, Impulse and Stroke Efficiency.

Muscle	Velocity (m·s^−1^)	
	Peak	Mean
Stroke Efficiency	−0.238	−0.426
Rate of Force Development (N·s^−1^)	0.656**	0.645**
Peak Upper Trunk Rotation to Stroke Side (°)	0.110	−0.050
Peak Lower Trunk rotation to the Stroke Side (°)	−0.684**	−0.567*
Impulse (Ns)	−0.088	−0.006

* denotes significance at p< 0.05, ** denotes significance at p< 0.01

Further significant correlations on the ipsilateral side were identified for the external oblique, characterised by significant positive relationships with mean (PA: *r* = 0.544) and peak (PA: *r* = 0.574; MA: *r* = 0.500) velocity. The peak and mean contralateral rectus abdominus displayed significant positive associations with mean velocity (PA: *r* = 0.562; MA: *r* = 0.651) and peak velocity (MA: *r* = 0.526). The peak and mean activation of the ipsilateral rectus abdominus exhibited significant positive associations with the mean kayak velocity (PA: *r* = 0.533; MA: r = 0.639). The contralateral rectus femoris was identified to have moderate significant positive correlations in both mean and peak activation with both mean and peak velocity. Conversely, the contralateral rectus femoris displayed moderate significant negative associations with lower trunk rotation to the stroke side (*r*< −0.560), for both mean and peak activation. This was also true for the ipsilateral eternal oblique, although strength of correlation was greater (*r*< −0.7). The ipsilateral external oblique also exhibited moderate significant correlations with the rate of force development, for both peak and mean activation (*r* = 0.560 and *r* = 0.587, respectively). The association with the rate of force development was also seen for the contralateral rectus abdominus (PA: *r* = 0.587; MA: *r* = 0.600), in addition to significant associations with mean (PA: *r* = 0.562; MA: *r* = 0.651) and peak (MA: *r* = 0.526) velocity (Table 3).

**Table 3:** Correlation coefficients between mean and peak muscle activation and velocity, rate of force production and trunk rotation.

Muscle	Velocity (m·s^−1^)				Rate of Force (N·s^−1^)		Peak Rotation to Stroke Side (°)			
	Peak		Mean				Upper Trunk		Lower Trunk	
	Peak (%MVC)	Mean (%MVC)	Peak (%MVC)	Mean (%MVC)	Peak (%MVC)	Mean (%MVC)	Peak (%MVC)	Mean (%MVC)	Peak (%MVC)	Mean (%MVC)
C-RF	0.621^*^	0.612^*^	0.678^**^	0.678^**^	0.319	0.263	0.337	0.276	−0.618^*^	−0.569^*^
I-RF	0.482	0.459	0.580^*^	0.562^*^	0.141	0.094	0.242	0.194	−0.407	−0.512
C-LD	0.038	0.044	0.024	0.018	−0.118	−0.197	0.464	0.545^*^	0.033	0.024
I-LD	0.074	0.050	0.178	0.213	0.215	0.115	−0.028	−0.130	0.055	0.191
C-EO	0.144	0.241	0.296	0.402	0.116	0.072	−0.530^*^	−0.405	−0.276	−0.244
I-EO	0.574^*^	0.500^*^	0.544^*^	0.497	0.560^*^	0.587^*^	0.060	0.188	−0.722^**^	−0.720^**^
C-RA	0.444	0.526^*^	0.562^*^	0.651^**^	0.587^*^	0.600^*^	−0.422	−0.437	−0.422	−0.422
I-RA	0.447	0.497	0.533^*^	0.639^**^	0.376	0.513^*^	−0.194	−0.148	−0.611^*^	−0.514

where C = Contralateral; I = Ipsilateral; RF = Rectus Femoris; LD = Latissimus Dorsi; EO = External Oblique; RA = Rectus Abdominus.

* denotes significance at *p*< 0.05, ** denotes significance at *p*< 0.01.

Assessment of the correlations between muscles also yielded interesting significant findings (Table 4), with the rectus abdominus demonstrating moderate to strong correlations with the rectus femoris, external obliques and between ipsilateral and contralateral sides. There were also strong significant positive associations between ipsilateral and contralateral rectus femoris; although the external obliques demonstrated a trend to associate with other muscles rather than in a pair. All comparisons between mean and peak activations of the same muscles demonstrated very strong associations (*r*> 0.9).

**Table 4:** Correlation coefficients between mean and peak muscle activation across all measured muscles.

C-RF	I-RF	C-LD	I-LD	C-EO	I-EO	C-RA	I-RA		
-	0.811**	0.260	0.443	−0.102	0.461	0.424	0.503*	P vs. P	C-RF
-	0.859**	0.319	0.514*	0.063	0.478	0.527*	0.648**	M vs. M	
0.965**	0.780**	0.302	0.416	−0.016	0.481	0.480	0.595*	P vs. M	
-	-	0.450	0.426	−0.015	0.456	0.285	0.585*	P vs. P	I-RF
-	-	0.479	0.429	0.271	0.574	0.412	0.759**	M vs. M	
0.887**	0.935**	0.509*	0.524*	0.129	0.488	0.368	0.691**	P vs. M	
-	-	-	0.041	−0.250	0.221	−0.421	0.188	P vs. P	C-LD
-	-	-	0.179	−0.224	0.176	−0.432	0.138	M vs. M	
0.249	0.394	0.962**	0.203	−0.179	0.179	−0.394	0.200	P vs. M	
-	-	-	-	0.012	−0.029	0.403	0.235	P vs. P	I-LD
-	-	-	-	0.162	−0.003	0.365	0.482	M vs. M	
0.514*	0.297	0.006	0.918**	−0.003	−0.006	0.429	0.362	P vs. M	
-	-	-	-	-	0.347	0.543*	0.768**	P vs. P	C-EO
-	-	-	-	-	0.128	0.502*	0.688**	M vs. M	
−0.052	0.112	−0.350	0.106n	0.945**	0.192	0.587*	0.689**	P vs. M	
-	-	-	-	-	-	0.469	0.645**	P vs. P	I-EO
-	-	-	-	-	-	0.498*	0.662**	M vs. M	
0.461	0.541*	0.200	−0.015	0.257	0.970**	0.519*	0.694**	P vs. M	
-	-	-	-	-	-	-	0.740**	P vs. P	C-RA
-	-	-	-	-	-	-	0.762**	M vs. M	
0.474	0.359	−0.462	0.341	0.467	0.459	0.985**	0.730**	P vs. M	
-	-	-	-	-	-	-	-	P vs. P	I-RA
-	-	-	-	-	-	-	-	M vs. M	
0.545*	0.685*	0.106	0.341	0.749**	0.588*	0.749**	0.956**	P vs. M	

where C = Contralateral; I = Ipsilateral; RF = Rectus Femoris; LD = Latissimus Dorsi; EO = External Oblique; RA = Rectus Abdominus; P = Peak Activation during stroke; M = Mean activation during the stroke.

* denotes significance at *p*< 0.05, ** denotes significance at *p*< 0.01.

## Discussion

Contradictory to the hypothesised association between trunk rotation and kayak velocity, grounded in previous work from this group (Brown et al., 2011) and that of Petrone et al. (2006), increased trunk rotation did not significantly associate with increased performance, as measured by kayak velocity. Conversely, a moderate negative association between lower trunk rotation to the stroke side and mean (*r* = −0.567) and peak (*r* = −0.684) velocity was identified, indicating that an increased kayak velocity was associated with a reduced lower trunk rotation. This was supported by significant negative associations between the ipsilateral external oblique and lower trunk rotation (PA: *r* = −0.722; MA: *r* = −0.720, *p* < 0.01) and significant positive correlations between ipsilateral external oblique activation and mean (PA: *r* = 0.544) and peak (PA: *r* = 0.574; MA: *r* = 0.500) velocity. The combination of these associations indicates that paddlers contract their ipsilateral external oblique to minimise the lower trunk rotation to the stroke side, increasing both peak intra stroke and mean velocity. Negative associations with lower trunk rotation were also observed in both the ipsilateral and contralateral rectus abdominus; although only the peak activation of the former was deemed to be significant (*r* = −0.611, *p*< 0.05). It can be proposed that the associations observed between lower trunk rotation and superficial abdominal musculature are used to stabilise the athlete and kayak during the paddle stroke.

Stabilising the lower trunk becomes of greater importance when effects of excessive trunk rotation on kayak motion are considered. As explained by Kemecsey (1986), during paddling technique the paddler rotates the trunk during the recovery and air work phases before transferring the weight onto the blade during the next stroke. The combination of this trunk rotation and shift in weight causes a moment to be applied to the kayak, resulting in a rotation of the kayak hull around its longitudinal axis. This motion of the kayak causes an increase in the wetted area of the kayak, consequently increasing the drag experienced by the kayaker, reducing the paddler’s velocity. This implication of trunk rotation aids in the explanation of the findings from the electrotorsiometer and lower trunk muscle activation. Consequently, the rotation of the entire trunk is not as important as previously indicated (Brown et al., 2011; Petrone et al., 2006). Moreover, the rotation produced in the lower trunk could be considered as a necessity to allow the blade recovery in preparation for the next catch, rather than a marker of improved performance. Instead, the rotation of the trunk that is required in the kayak stroke, as identified by Kemecsey (1986), Petrone et al. (2006) and Brown et al. (2011), shows that it should occur in the upper trunk, to allow the contribution of the larger trunk muscles (i.e., latissimus dorsi and trapezius) and reduce the unwanted longitudinal rotation of the kayak. However, no significant correlations between upper trunk rotation to the stroke side and velocity were identified. Therefore, the rotation of the trunk, which to date has been promoted as being important, could be suggested to be a necessity to maintain the paddle in the correct orientation to optimise the benefits of the wing blade paddles, rather than a determinant of performance. Resultantly, the hypothesised association between increased trunk rotation and performance was rejected.

Examination of the trunk and leg musculature adds further insight into the musculature that drives performance in sprint kayaking. Specifically, significant correlations between the ipsilateral external oblique and mean (*r* = 0.515, *p*< 0.05) and peak (*r* = 0.518, *p*< 0.05) velocity were identified. However, it should be noted that during paddling, peak levels of activation reached 250% MVC indicating that the MVC protocol was not sufficient to elicit a maximal contraction from the external obliques. Therefore, the MVC for the external oblique may still serve as a reference contraction for comparison, as the protocol was identical for all participants, however, recommendations from these findings would have to be made with caution. No issues were experienced in the MVC for the rectus abdominus; the data from which a significant positive correlation between mean velocity and the contralateral rectus abdominus (*r* = 0.598, *p*< 0.05) was displayed. Moreover, positive associations between trunk musculature and knee extensors during paddling highlighted a significant moderate to strong interaction between the rectus abdominus, rectus femoris and external obliques. The ipsilateral external oblique demonstrated significant associations with the contralateral and ipsilateral rectus femoris and rectus abdominus (Table 2), while the contralateral rectus femoris demonstrated significant moderate positive association with peak and mean velocity (*r* = 0.621, *r* = 0.678, *p*< 0.05). These findings corroborate the importance of limiting lower trunk motion, as an isometric contraction of the ipsilateral external oblique will limit any rotation to the stroke side, with the contralateral rectus abdominus contracting to aid stability in the lower trunk. Furthermore, the contralateral rectus femoris activation is likely to be aiding a hip flexion pulling against the footplate to stabilise the motion, as the contralateral knee flexes during the paddle stroke (Kemecsey, 1986), which could be seen in the corroborating significant association between the rectus femoris and the rectus abdominus (*r* = 0.527). These findings fall in line with those indicative in other sports (Kibler et al., 2006) and underpin the findings of Davidek et al. (2018), who established the value of dynamic neuromuscular stabilisation in paddling force production, across a cohort of high performance sprint kayakers.

This study’s findings are not without their limitations, as the dynamic nature of the sprint kayak paddling technique may have influenced the measures. This could have been a factor in the excessively high scores reported for the external obliques, though this could also have been influenced by the manual nature of the resistance provided during the maximal voluntary contraction protocol. Moreover, the measures of axial rotations of the lower trunk may have been influenced by extension of the legs and motion in the pelvis. However, while these may influence the relative measures reported, in the context of performance, limiting the rotation of the lower trunk to the stroke side appears to be fundamental to higher kayak velocity. Therefore, the reduced rotation in the lower trunk is a novel indicator of performance previously unidentified due to the dependence on on-ergometer paddling as the primary methodology of investigation.

From the findings, the experimental hypotheses were rejected, as increased trunk rotation and increases in ipsilateral latissimus dorsi and contralateral external oblique activation displayed no relationship with kayak velocity. Conversely, stabilising the lower trunk has been demonstrated to be important in producing (inter stroke peak) and maintaining (mean) kayak velocity. This process is characterised by limiting the rotation of the lower trunk to the ipsilateral side, by contracting the ipsilateral external oblique and contralateral rectus abdominus during the paddle stroke. The reduction in lower trunk rotation could limit the increase in the wetted surface area experienced, due to the longitudinal rotation of the kayak during the stroke and the resultant increase in drag experienced, minimising the reduction in kayak velocity; although further evidence would be needed to empirically establish this. It is therefore important that during off-water physical training the lower trunk muscles are addressed, alongside the traditional strength training regimes employed by sprint kayaking coaches.
